# Effect of Cathodic Polarisation Switch-Off on the Passivity and Stability to Crevice Corrosion of AISI 304L Stainless Steel

**DOI:** 10.3390/ma14112921

**Published:** 2021-05-28

**Authors:** Varvara Shubina Helbert, Andrei Nazarov, Flavien Vucko, Nicolas Larché, Dominique Thierry

**Affiliations:** French Corrosion Institute, 220 Rue Pierre Rivoalon, 29200 Brest, France; nazarovandrei@neuf.fr (A.N.); flavien.vucko@institut-corrosion.fr (F.V.); nicolas.larche@institut-corrosion.fr (N.L.); dominique.thierry@institut-corrosion.fr (D.T.)

**Keywords:** cathodic polarisation, hydrogen, stainless steel, crevice corrosion, SKP, passivation, TDA, electrochemical measurements

## Abstract

The effects of cathodic polarisation switch-off on the passivation of AISI 304L stainless steel in air and its crevice corrosion susceptibility in 3.5 wt.% NaCl aqueous electrolyte were investigated. Scanning Kelvin probe (SKP) data showed that the oxide film is significantly destabilised and the rate of steel passivation in air is slowed down. Thermal desorption analysis (TDA) highlighted that hydrogen absorption is proportional to the applied cathodic current density. A special crevice corrosion set-up was designed to realise simultaneous reproducible monitoring of potential and galvanic current to study the impact of prior cathodic polarisation on crevice corrosion onset.

## 1. Introduction

The technique of impressed current cathodic protection (ICCP) was introduced in 1928 for the protection of steel gas pipelines in the United States, and it is now widely used for many industrial applications [1]. Indeed, ICCP stations are widely used to increase the service life of large structures, such as oil and gas offshore pipelines and storage tanks, where the use of galvanic anodes is not possible [2]. It is important to underline that due to electrical continuity, stainless steels typically used as critical components of offshore structures are also subjected to impressed current. In addition, the impressed current can be switched off due to various maintenance procedures at the cathodic stations, leaving the structures at the free corrosion potential [3]. Thus, it is important to study how cathodic polarisation affects various properties of stainless steel. 

During cathodic polarisation, hydrogen, as a product of cathodic water reduction, diffuses inside the stainless steel and thus can modify the surface composition [4], induce phase transformation [5] or alter the corrosion resistance and even the mechanical properties [6]. In the pioneer works of Oriani [4,7], it was reported that passive iron and nickel become remarkably less resistant to pitting corrosion after cathodic charging. Hydrogen is a strong reducing agent and thus can alter the chemical composition of the passive film on iron by reducing metal oxides [8]. It was also highlighted that this ability to react with O^2−^ and OH^−^ in the passive film decreases the apparent diffusivity of hydrogen in this layer compared to the metal lattice [4]. Therefore, hydrogen absorbed in the steels and the passive films during cathodic polarisation may affect the stability of the passive layer and, consequently, the susceptibility of steel against corrosion. 

Regarding stainless steels, a decrease in the corrosion resistance after hydrogen charging was reported for austenitic [9,10,11,12,13,14,15], martensitic [16] and duplex [17,18,19] stainless steels.

Yashiro et al. observed that for AISI 304 (UNS 30400), the permeated hydrogen shortened the induction periods for pitting corrosion using a Devanathan–Stachurski cell (charging side: 0.1 M Na_2_SO_4_) and polarisation curves obtained from the detection side (0.1 M and 0.5 M NaCl) [9]. Wang et al. observed weaker intensities of all Fe compounds (Fe^3+^, Fe^2+^ oxides and hydroxides) and a stronger intensity of metallic iron for hydrogen-charged AISI 304 in 0.5 M H_2_SO_4_ + 0.25 g/L thiourea [20]. As suggested in the study of Cui et al. [21], an increase in metallic iron indicates that the passive film becomes thinner compared to uncharged AISI 304 due to the destabilisation caused by hydrogen. Hua et al. tried to understand the hydrogen local transport behaviour and highlighted a heterogeneous hydrogen distribution after thermal hydrogen charging of AISI 304 in 82 MPa H_2_ at 573 K for 250 h [11]. The investigation of AISI 316 (UNS S31603) by Ningshen et al. showed an increase in the passive current, accompanied by a decrease in the pitting potential after hydrogen pre-charging in 0.1 N NaOH + 250 ppm As_2_O_3_ [14]. The authors linked the breakdown of the passive film with hydrogen atoms due to the proton de-insertion reaction (electro-oxidation) and reduction of Fe^3+^ to Fe^2+^. The synergy between stress and hydrogen induced at −10 mA cm^−2^ for 2 h in 0.5 M H_2_SO_4_ + 250 ppm As_2_O_3_ of AISI 304 was demonstrated in [12].

According to literature data, it seems clear that switching off cathodic protection can lead to localised corrosion of stainless steel. However, despite numerous studies dedicated to this issue, there still large gaps in the knowledge on the effect of absorbed hydrogen on the initiation of localised corrosion of stainless steel. Moreover, in most of the works in this field, hydrogen was introduced into the steel using extremely high current densities and using model-charging solutions far from practical situations (hydroxides, borates, acids, etc.). Therefore, it is important to study the impact of hydrogen on localised corrosion of stainless steel under more relevant conditions, for instance, marine environments. 

In the present investigation, the impact of prior cathodic polarisation (CP) on the crevice corrosion of AISI 304L (UNS 30403) stainless steel was studied in 3.5 wt.% NaCl. The influence of three cathodic current densities was studied with regards to the susceptibility of AISI 304L stainless steel to crevice corrosion. To investigate the impact of cathodic polarisation switch-off on the initiation of crevice corrosion, simultaneous monitoring of potential and galvanic current was carried out in a specially designed crevice assembly cell. Scanning Kelvin probe (SKP) analysis was used to study the impact of hydrogen induced by cathodic polarisation on the passivation in air, and thermal desorption analysis (TDA) was carried out to quantify the total hydrogen content and to analyse its evolution after various desorption times.

## 2. Materials and Methods

### 2.1. Materials and Preparation

The composition of the austenitic AISI 304L (UNS S30403) stainless steel used in this study is reported in Table 1. The samples were mechanically ground down to 1200 grit SiC emery paper. After polishing, the samples were degreased with acetone, rinsed in deionised water and dried with compressed air. 

### 2.2. Electrochemical Techniques

#### 2.2.1. Cathodic Polarisation

AISI 304L samples were cathodically polarised in 3.5 wt.% NaCl aqueous solution, simulating exposure of steel constructions in seawater under conditions of cathodic protection. The concentration of 3.5 wt.% NaCl was used to represent the salinity level of seawater (35 ppt = 3.5 wt.% NaCl) [22]. Despite the fact that other ions, such as K^+^, Mg^2+^ and SO_4_^2–^, are also present in seawater, Na^+^ and Cl^−^ ions correspond to 91% of all ions [22]. AISI 304L was used as a working electrode (WE). The counter-electrode (CE) with a surface area of approximately 300 cm^2^ was a rectangular wire of Ti/MMO (mixed-metal oxide KERAMOX^®^ from Evoqua MAGNETO, Schiedam, The Netherlands) commonly used in ICCP due to it corrosion resistance under acid conditions typical for oxygen- and chlorine-evolving environments [23]. The reference electrode (REF) was a saturated calomel electrode (SCE; Hg/Hg_2_Cl_2_, E = +0.241 V vs. standard hydrogen electrode (SHE)). Cathodic polarisation was carried out galvanostatically at three current densities of −10 µA cm^−2^, −2 mA cm^−2^ and −10 mA cm^−2^ and a duration of 100 ks (~27.8 h). The low current density (−10 µA cm^−2^) corresponds to the current value recommended for cathodic protection in seawater according to the standard DNV RP 401 [24]. The higher current values, −2 and −10 mA cm^−2^ used previously in [25], were used in this study. 

#### 2.2.2. Crevice Corrosion Testing

**Figure 1:**
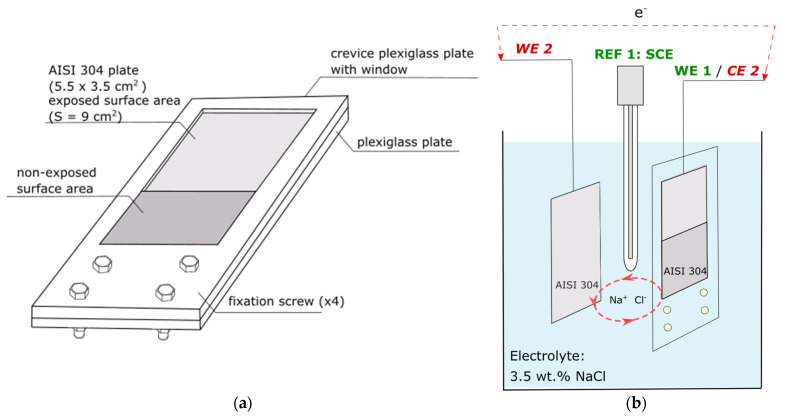
(**a**) Schematic representation of the crevice corrosion assembly and (**b**) experimental set-up for OCP and galvanic current recordings after cathodic polarisation switch-off with a cathodically pre-polarised AISI 304L plate used as a working electrode (WE 1: embedded in the crevice assembly) for OCP recording and as a counter-electrode (CE 2) for galvanic current measurements, a saturated calomel electrode (REF 1: electrode-SCE) for OCP measurements and another AISI 304L plate (WE 2: plate without a crevice) introduced for galvanic current recordings.

A schematic representation of the crevice corrosion assembly used in this study is shown in Figure 1a. To generate a crevice in a reproducible way, an AISI 304L sample (55 × 35 mm^2^, l = 0.5 mm) was fixed between two transparent plastic plexiglass plates using 4 fixation screws at a gap of 0.5 mm (Figure 1a). The so-called crevice plexiglass plate contained a window to expose one side of the steel sample (S = 9 cm^2^) to 3.5 wt.% NaCl electrolyte. A low-aerated area of the sample was created inside the gap to localise the crevice corrosion phenomenon. At first, cathodic polarisation (described in detail in the previous section) was applied on the AISI 304L sample mounted in the crevice assembly. After CP switch-off, the open circuit potential (OCP) and the current were monitored simultaneously to investigate the effect of cathodic polarisation on the time of crevice corrosion initiation, as represented in Figure 1b, using a multichannel Origaflex OGR-500 (Origalys, Lyon, France). For OCP measurements, the cathodically charged AISI 304L sample (WE 1) and a reference electrode (REF1: SCE) were used. To perform galvanic current measurements, a new AISI 304L sample (WE 2) was introduced in the system at the end of the cathodic polarisation (Figure 1b). The measurements of the potential and the galvanic current made possible the estimation of the incubation time of crevice corrosion. This was compared with visual observations of the corrosion in situ through the transparent crevice plexiglass plate. The electrochemical results are shown for a single sample but were obtained from at least three separate experiments.

After exposure, the specimens were removed from the crevice assembly and rinsed in deionised water and ethanol. An SU3500 scanning electron microscope (SEM; Hitachi, Tokyo, Japan) was used to analyse the morphology of the corrosion in creviced zones. Measurements of the corrosion attack depth were performed by the focalisation technique on each corroded sample after crevice corrosion tests and corrosion product removal with a Leica DMC 2900 optical microscope. The technique consists of focalising on the bottom of the deepest point of the corroded area and then on the non-corroded surface. The height difference was measured using a digital micrometre comparator, Mitutoyo ID-C112B model, set on the microscope.

### 2.3. Thermal Desorption Analysis

Hydrogen quantifications were performed using a Galileo G8 katharometer (Bruker, Billerica, MA, USA). Thermal desorption analysis (TDA) was conducted using a constant heating rate of 1 °C/s from room temperature up to 850 °C in a nitrogen atmosphere, accompanied by hydrogen release and its quantification. Prior to measurements, the calibration of the instrument was performed with a calibration gas (helium). AISI 304L specimens (20 × 60 × 0.5 mm^3^) were hydrogen-charged at −10 µA cm^−2^, −2 mA cm^−2^ and −10 mA cm^−2^ for 100 ks (~27.7 h) in 3.5 wt.% NaCl. TDA was carried out on the samples within 10 min after the charging. Some specimens were also placed in a desiccator at room temperature for 24, 48 or 168 h prior to TDA for desorption of hydrogen and to quantify the desorption rate of diffusible hydrogen. The diffusible hydrogen was defined as the amount of hydrogen that was able to desorb at room temperature, corresponding to the hydrogen peaks observed below 450 °C for the selected heating rate.

### 2.4. Scanning Kelvin Probe Analysis

In this study, a height-controlled SKP instrument (vibrating capacitor) from Wicinski & Wicinski GbR (Erkrath, Germany) was used. The reference electrode was a needle (NiCr alloy) with a diameter of 100 µm, and the distance to the working electrode surface was close to 50 µm. The SKP needle was attached to a permanent magnet and vibrated at a frequency of 1 kHz, and the AC baking potential frequency was 10 Hz. Before and after measurements, the potential of the probe was calibrated above the saturated Cu/CuSO_4_ electrode. However, for more clarity, all SKP measurements results were recalculated versus the saturated calomel electrode (SCE). Cathodic polarisation was performed locally in order to compare the potential of the cathodically polarised surface with the potential of the initial one. AISI 304L specimens (40 × 40 × 0.5 mm^3^) with an exposed circular surface of 0.16 cm^2^ were hydrogen-charged at −10 µA cm^−2^, −2 mA cm^−2^ and −10 mA cm^−2^ for 100 ks (~27.8 h) in 3.5 wt.% NaCl. The first potential distribution scan was carried out ex situ and started within 10 min after cathodic polarisation (t_0_). Then, the specimens were left in laboratory air at room temperature for 24, 48, 72, 168 and 672 h prior to SKP scans to investigate the surface potential evolution due to interaction with air and passivation. SKP measurements were performed in laboratory air at ambient temperature and humidity (50–60% RH and 20–22 °C). OriginPro 9.0 software was used for data analysis and graphing of 3D surface color maps.

## 3. Results and Discussion

### 3.1. Potential Monitoring during Cathodic Polarisation

**Figure 2:**
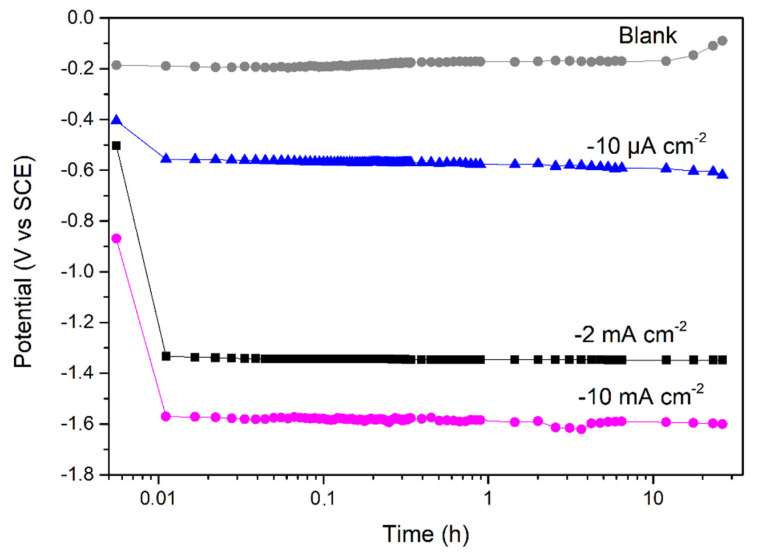
Monitoring of potential evolution in 3.5 wt.% NaCl without (blank) and with cathodic polarisation at −10 µA cm^−2^, −2 mA cm^−2^ and −10 mA cm^−2^ for 100 ks (~27.8 h).

Figure 2 shows the recorded potential during cathodic polarisation in 3.5 wt.% NaCl using current densities of −10 µA cm^−2^, −2 mA cm^−2^ and −10 mA cm^−2^ and a charging duration of 100 ks. Chronopotentiometric data presented in Figure 2 clearly demonstrate an abrupt potential shift in the cathodic domain at all current densities. The potential values were depressed to about −0.64, −1.37 and −1.60 V vs. SCE at the three applied current densities of −10 µA cm^−2^, −2 mA cm^−2^ and −10 mA cm^−2^, respectively. From Pourbaix thermodynamic calculations, oxides of iron and chromium are reduced at potentials more negative than −0.37 and −1.34 V_SCE_, respectively [26]. Thus, we can suppose that partial or completed reduction of the surface oxide film occurred. Additionally, cathodic polarisation leads to thermodynamic non-stability of water molecules, creating atomic hydrogen at the surface of the cathode (Equation (1)), where H(ads) is adsorbed hydrogen. Atomic hydrogen recombines or electrochemically oxidises, forming molecular hydrogen H_2(gaz)_ in (Equation (2)). In contrast, atomic hydrogen can be absorbed (H_(abs)_) in the subsurface of the alloy (Equation (3)) [27].

H_2_O + e^−^ → H_(ads)_ + OH^−^ (1)

H_(ads)_ +H_(ads)_ → H_2 (gaz)_ (2)

H_(ads)_ → H_(abs)_ (3)

In contrast, for the unpolarised sample (blank in Figure 2), the potential slowly increased from −0.19 to −0.06 V vs. SCE due to the growth of a surface oxide film [28]. 

### 3.2. TDA Quantification of Hydrogen Content

**Figure 3:**
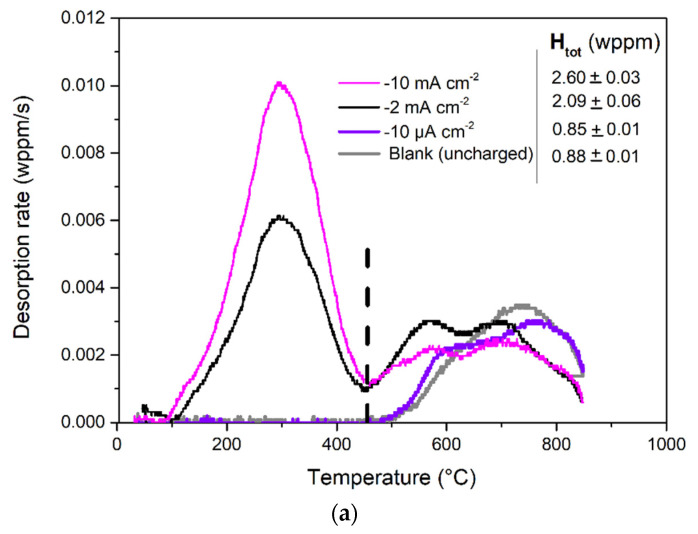

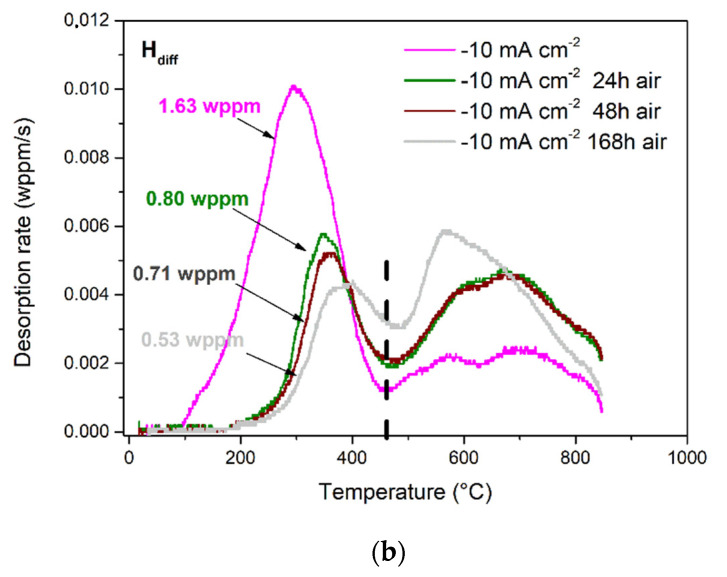
Desorption curves obtained from TDA measurements of AISI 304L stainless steel for (**a**) uncharged samples (blank) and samples within 10 min after cathodic polarisation in 3.5 wt.% NaCl for 100 ks at −10 µA cm^−2^, −2 mAcm^−2^ and −10 mA cm^−2^ and (**b**) after cathodic charging at −10 mA cm^−2^ and exposure to air for 24, 48 and 168 h. The total hydrogen content (H_tot_) values are indicated in (**a**), and the diffusible hydrogen content (H_diff_) from the first peak deconvolution are given in (**b**).

To quantify the hydrogen induced by cathodic charging, TDA was performed for all three studied current densities at a fixed charging time of 100 ks (~27.8 h). TDA measures the hydrogen desorption rate when a sample containing hydrogen is heated at the constant rate of 1 °C/s. The TDA spectra with the total hydrogen content are given in Figure 3a. Furthermore, the integration of the spectra provides the amount of desorbed hydrogen from the samples in the considered temperature range. The total hydrogen content was defined as the amount of desorbed hydrogen up to the maximum tested temperature of 850 °C. The obtained spectra for the blank (uncharged) sample showed one peak at high temperature, attributed to the initially (irreversibly) trapped hydrogen at material heterogeneities such as vacancies, precipitates and interphases [29]. The thermal desorption spectrum for the sample after cathodic charging at −10 µA cm^−2^ presents the same trend as the blank. The hydrogen content was evaluated at 0.85 ± 0.01 wppm (0.88 ± 0.01 wppm was detected for the blank). For the samples charged at −2 and −10 mA cm^−2^, cathodically induced hydrogen significantly modified TDA spectra and an additional peak of diffusible hydrogen appeared in the low-temperature domain (100–400 °C). The total hydrogen content thus increased up to 2.09 ± 0.06 and 2.60 ± 0.03 wppm for −2 and −10 mA cm^−2^, respectively. 

Figure 3b displays the thermal desorption spectra after charging at −10 mA cm^−2^ and after various desorption times in air. As shown, complete desorption of the diffusible hydrogen content was not achieved even after 168 h of desorption at room temperature. Two main phenomena were also highlighted from these results: (i) the decrease in the diffusible hydrogen peak intensity was proportional to the desorption time (values given after peak deconvolution; cf. Figure 3b) and (ii) the hydrogen in the material was modified and redistributed with desorption time, especially in irreversible trap sites (cf. increase and modification of the peak at >500 °C in Figure 3b). The tendency observed from the spectra given in Figure 3b also emphasises the fast desorption kinetics during 24 h of desorption and its further stabilisation. Thus, it could be assumed that after cathodic charging, only part of the diffusible hydrogen is released from the steel, affecting the surface, and another part is deeply trapped in the microstructure.

### 3.3. Scanning Kelvin Probe Analysis

**Figure 4:**
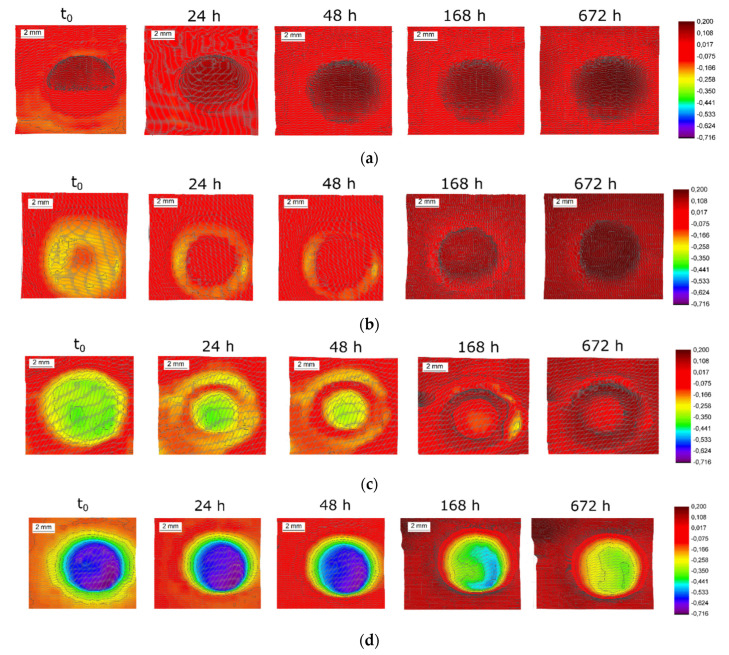
SKP potential maps (V vs. SCE) of AISI 304L stainless steel (S_circle_ = 0.16 cm^2^) for (**a**) sample after exposure to 3.5 wt.% NaCl for 100 ks at the OCP and samples after cathodic polarisation at −10 µA cm^−2^ (**b**), −2 mA cm^−2^ (**c**) and −10 mA cm^−2^ (**d**) in 3.5 wt.% NaCl for 100 ks. SKP maps were recorded in air at t_0_ (within 10 min after sample preparation) and after exposure to air for 24, 48, 168 and 672 h.

SKP was used to investigate the impact of cathodic polarisation on the potential distribution across the AISI 304L surface and its evolution over time. Figure 4 displays the series of SKP potential maps recorded in air after exposure to 3.5 wt.% NaCl aqueous electrolyte for 100 ks at the open circuit potential (OCP) (Figure 4a) and at the cathodic polarisation at different current densities (Figure 4b–d). SKP potential distribution maps clearly demonstrated a circular feature (S_circle_ = 0.16 cm^2^) that corresponds to the surface exposed to the electrolyte and cathodically polarised. The first potential distribution scan was carried out within 10 min after cathodic polarisation (t_0_). 

In the case of exposure at the OCP, the circular region revealed an increased potential (Figure 4a) compared to the surrounding area non-exposed to the electrolyte, with a difference of about 0.20 V (Δ_t0_). It is known that the potential of stainless steel is sensitive to the oxide film thickness [30]. Thus, it is possible that long-time exposure to the NaCl electrolyte led to a thickening of the surface oxides. According to Olsson and Landolt, chloride can be adsorbed or incorporated during the passive film growth in the presence of chloride anions [28]. As a result of the thickening of the surface oxide after 48 h, the potential of the overall surface uniformly increased by 0.10 ± 0.03 V. After 48 h of exposure, the process became slower, and no significant evolution of the potential was observed (Δ_168h_ = Δ_672h_ = 0.10 V). A similar effect was observed in the electrolyte during monitoring of the OCP for the unpolarised sample (cf. blank in Figure 2).

SKP maps given in Figure 4b–d were measured after cathodic polarisation of AISI 304L at three current densities, −10 µA cm^−2^, −2 mA cm^−2^ and −10 mA cm^−2^, for 100 ks. Based on the obtained maps at t_0_, it can be clearly stated that cathodic polarisation shifts the surface potential downwards. Additionally, an increase in the current density shifts the potential to more negative values. This can be the result of oxide film reduction by the imposed current and accumulated subsurface hydrogen. SKP maps measured after 24, 48, 168 and 672 h of exposure to air show a potential increase likely due to the growth of the oxide film, as shown in [25]. Two factors influence the oxide thickness: (1) the reduction of the oxide by effused atomic hydrogen and (2) the oxide growth due to oxidation by air. However, the stainless steel surface polarised at −10 mA cm^−2^ did not recover even after 672 h (1 month) of air exposure, and the potential of charged area remaining around −0.30 V vs. SCE. Hence, cathodic polarisation at high current densities significantly inhibits passivation and irreversibly modifies the oxide film.

Potential non-uniformities were observed in SKP maps (Figure 4b,c) that relate to properties of the formed oxides. Hydrogen can laterally diffuse out from the polarised areas. The hydrogen reduces the native oxide film and creates a low-potential ring in the maps. This effect becomes visible after 24 h of exposure to air. In the case of polarisation at −10 µA cm^−2^, the area under the O-ring with a potential of around −0.18 V vs. SCE was found to be a zone with an increased subsurface hydrogen concentration and could be a preferential zone for crevice corrosion initiation [25].

SKP potential distribution maps highlighted that passivation by oxygen in air does not occur rapidly and homogeneously after cathodic polarisation switch-off. Thus, cathodic polarisation irreversibly changes the oxide film, decreasing the potential drop in it. It is well known that hydrogen induced by cathodic polarisation can modify the oxide film composition by reducing the Fe^3+^ to Fe^2+^ [31] and by the reaction of H^+^ ions with O^2−^ and OH^−^ species [8]. Moreover, the passivation ability also decreased due to continuous hydrogen outward diffusion. Therefore, if cathodic polarisation is switched off and AISI 304L is left at the OCP in the chloride-containing electrolyte, the risk of passive film breakdown and localised corrosion initiation becomes significant.

### 3.4. Crevice Corrosion Tests

It was highlighted in previous works that the sensitivity of AISI 304L to crevice corrosion increases after cathodic polarisation with an increase in the current density and charging duration [25]. In this work, the crevice assembly (cf. Figure 1a) was developed to quantify the impact of cathodic pre-polarisation.

**Figure 5:**
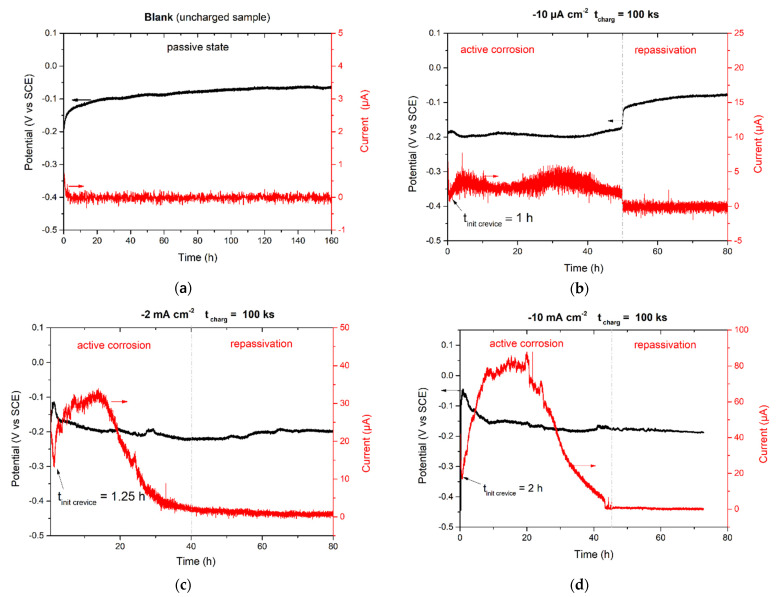
Open circuit potential and galvanic current recordings for AISI 304L stainless steel in 3.5 wt.% NaCl aqueous electrolyte in ac revice assembly (**a**) without CP (blank) and after cathodic polarisation for 100 ks at (**b**) −10 µA cm^−2^, (**c**) −2 mA cm^−2^ and (**d**) −10 mA cm^−^^2^.

Figure 5 shows potential and current measurements for the AISI 304L samples embedded in a crevice assembly without (Figure 5a) and with preliminary cathodic polarisation (cf. Figure 5b–d). Figure 5a demonstrates a potential shift to more noble potentials during the experiment and shows passivation and no crevice corrosion. The same behaviour was found previously for AISI 304L without crevice assembly (see the previous section; Figure 2, blank). It should be noted that even after 160 h, the OCP was not at steady-state conditions and continued to increase slowly, showing the evolution of the passive film [32,33]. In addition, the galvanic current was close to zero (Figure 5a) during the whole experiment, highlighting the fact that of crevice corrosion was not initiated. However, some current instabilities were observed. These could probably be related to the initiation, growth and repassivation of metastable pits upon the surface [34]. However, metallographic examinations have shown the absence of localised corrosion.

**Figure 6:**
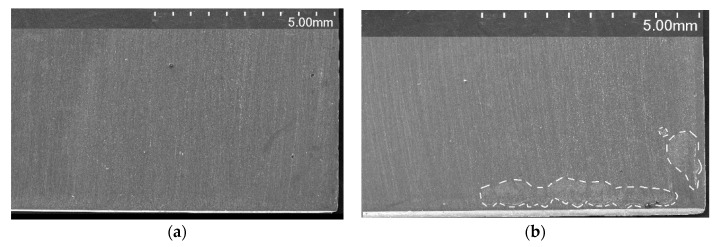

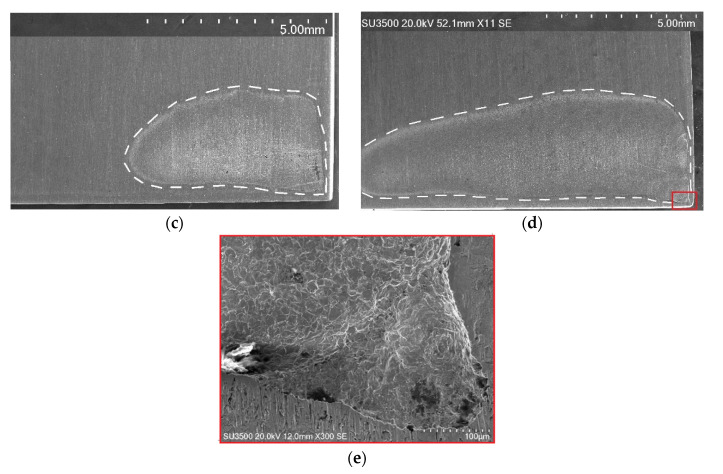
SEM micrographs (SU3500; Hitachi, Tokyo, Japan) of AISI 304L samples after crevice corrosion tests in 3.5 wt.% NaCl: (**a**) blank (uncharged sample); CP at (**b**) −10 µA cm^−2^, (**c**) −2 mA cm^−2^ and (**d**) −10 mA cm^−2^ for 100 ks; and (**e**) the area inside the crevice (cf. red rectangle from (**d**)). The crevice corrosion zones were found on the plate corners for cathodically polarised samples.

Figure 5b shows the same type of data but after previous cathodic polarisation at a current density of −10 µA cm^−2^. After cathodic polarisation switch-off, the OCP curve showed a potential increase as was the case for the unpolarised sample. However, after roughly 1 h, the potential started to drop abruptly, accompanied by a current rise. This may indicate a start of corrosion due to the permanent breakdown of the passive film [32]. Thus, the incubation time that was needed to initiate crevice corrosion after cathodic charging was approximately 1 h. In this experiment, the anodic current increased to 5 µA, showing anodic dissolution of the sample in the assembly (cf. secondary electron (SE) micrograph, Figure 6b). The potential was more negative relative to the non-polarised sample (Figure 5a). However, after exposure for 50 h, the potential increased and the current vanished. This time corresponds to the repassivation of the crevice.

Increasing the current density of cathodic pre-polarisation to −2 and −10 mA cm^−2^ increased the galvanic current to 30 and 80 µA, respectively (cf. Figure 5c,d). The potential starts to decrease significantly after 1–2 h of cathodic polarisation switch-off, accompanied by a current rise, showing crevice corrosion onset [32]. Then, the potential remains negative during exposure. However, with increasing time of exposure, the galvanic current significantly decreased due to partial passivation of the electrode. After sample removal from the crevice cell, it was visually confirmed that corrosion started and propagated in the crevice zone, designed by the crevice assembly. The crevice corrosion was found close to the corners for all the samples, as shown in Figure 6a–d (zones with a contour in the dashed line). The surface affected by crevice corrosion attacks increased with an increase in the density of the applied cathodic current and corresponds well to the measured galvanic current in Figure 5. The SEM micrograph in Figure 6e refers to the zone identified by the red rectangle in Figure 6d. It clearly shows a zone of anodic dissolution (crevice) with the maximum depth of attack on the sample corner and corrosion product deposits. The area inside the crevice is characterised by distinguished austenitic grains. Metallography analysis showed the depth of corrosion attacks for samples after cathodic polarisation at −2 and −10 mA cm^−2^ to be 21 ± 9 and 63 ± 8 µm, respectively, after 80 h of exposure.

The crevice corrosion process is mainly due to the spatial separation of two electrochemical reactions [35]:

(i) the cathodic reaction of oxygen reduction outside the crevice mouth:

O_2_ + 2H_2_O + 4e^−^ → 4OH^−^ (4)

(ii) the anodic reaction of iron and chromium dissolution inside the crevice mouth:

Fe → Fe^2+^ + 2e^−^ (5)

Cr → Cr^3+^ + 3e^−^ (6)

A differential aeration is created: the cathode (outside the crevice) is continuously supplemented by the oxygen, while the oxygen diffusion and reduction rate is significantly reduced at the anode (inside the crevice). Thus, magnetite is a typical corrosion product found in the crevices due to insufficient transport of oxygen [36]. This spatial separation of cathode and anode leads to the build-up of a localised corrosion cell, and the current flow creates an IR drop stabilising the galvanic cell. As stated by Pickering [37,38], crevice corrosion abruptly starts when a potential difference is established between the inside and the outside of the crevice.

The initiation of crevice corrosion of stainless steels in a neutral chloride solution containing dissolved oxygen is characterised by a local breakdown of the passive film inside the crevice [39]. This breakdown is marked by a drop in the corrosion potential, as observed in Figure 5b–d. However, the passive film outside the crevice remained intact. Once the initiation of corrosion inside the crevice happened, the propagation of corrosion started rapidly [35]. 

Experimental data of the present work showed that cathodically pre-polarised AISI 304L is subjected to crevice corrosion initiation after 1–2 h of cathodic polarisation. It was found that the rate of corrosion increased with the amount of hydrogen absorbed by the stainless steel during the polarisation. It is important to highlight that the activating effect of hydrogen seems to vanish after 40–50 h of exposure (Figure 5b–d). The galvanic current decreased to zero due to hydrogen depletion at the subsurface. However, this time did not depend on the amount of cathodic electricity passed and the hydrogen concentration in the sublayer. Probably the time needed for passivation is related to the time of effusion that is approximatively similar for different concentrations of hydrogen. Indeed, the same cathodic polarisation time (~27.8 h) was applied to all the samples; thus, the depth penetration of hydrogen and the effusion path were approximatively the same. 

The changes in the chemical composition of the passive film due to the presence of diffusible hydrogen could also be a reason of the localised corrosion onset [25]. The passive layer became less resistant because it was partially reduced due to the cathodic charging. These two contributions, previously applied cathodic current (i) and hydrogen desorption (ii) from the bulk, push the material sensitivity to crevice corrosion. It was also highlighted that hydrogen-charged steel surfaces consume more oxygen when compared to uncharged steel surfaces [40,41,42]. This is the result of strong interaction of atomic hydrogen with oxygen. Consequently, the hydrogen released by charged stainless steel accelerates the deoxygenation of the crevice solution and promotes the initiation and propagation of crevice corrosion. 

## 4. Conclusions

1. The corrosion stability is significantly altered by previous cathodic polarisation. The surface potential measured by a scanning Kelvin probe in air shifts negatively with an increase in the applied cathodic current density. The oxide films show a low potential drop. It was demonstrated that effusion of hydrogen from the bulk inhibits oxide film formation and alloy passivation.
2. TDA highlighted that the amount of diffusible hydrogen increases with the applied current density. A part of diffusible hydrogen presented fast desorption kinetics during 24 h in air, followed by stabilisation and redistribution in the material.
3. The cathodically polarised AISI 304L steel surface shows susceptibility to rapid initiation of crevice corrosion that was found by measuring the OCP and galvanic current in a specially designed crevice assembly. Accumulated subsurface hydrogen promotes the local breakdown of the passive film and significantly reduces the time needed to initiate crevice corrosion.

## Figures and Tables

**Table 1:** Nominal composition of the austenitic AISI 304L (UNS S30403) stainless steel (wt.%).

Fe	C	Si	Mn	P	S	Cr	Ni
Balance	Max. 0.03	0.6	1.5	Max. 0.045	Max. 0.03	18.5	9.5

## Data Availability

The data presented in this study are available on request from the corresponding author.
